# A Mobile App to Promote Adapted Exercise and Social Networking for People With Physical Disabilities: Usability Study

**DOI:** 10.2196/11689

**Published:** 2019-03-19

**Authors:** Byron Lai, Jereme Wilroy, Hui-Ju Young, Jennifer Howell, James H Rimmer, Tapan Mehta, Mohanraj Thirumalai

**Affiliations:** 1 UAB/Lakeshore Research Collaborative School of Health Professions University of Alabama at Birmingham Birmingham, AL United States; 2 Department of Health Services Administration University of Alabama at Birmingham Birmingham, AL United States

**Keywords:** exercise, telehealth, rehabilitation, mHealth

## Abstract

**Background:**

People with physical disabilities (PWD) experience several unique challenges that prevent them from participating in onsite exercise programs. Although mobile apps can provide a ubiquitous channel for delivering convenient exercise services within the community, no exercise apps have been designed for people with disabilities who experience certain functional limitations.

**Objective:**

The aim of this study was to examine the usability of a mobile exercise app in PWD.

**Methods:**

A sequential explanatory mixed-method design was used to holistically test usability in 4 core areas: *effectiveness* (ie, ease of use), *efficiency* (ie, operation speed), perceived *satisfaction*, and *usefulness.* Participants completed 7 face-to-face usability tasks and 1 structured interview. Equipment included a computer tablet that came preinstalled with the exercise app. The app included exercise videos that focused on several components of fitness: aerobic capacity, muscular strength, functional strength or balance, and range of motion. The app contained 3 different versions of the exercise program: (1) a program for people with the ability to use the upper and lower limbs, (2) a seated program for people with the ability to use only upper limbs, and (3) a program designed for people with hemiparesis. The app also included educational resources in the form of infographics aimed at addressing key social cognitive theory constructs included social support, outcome expectancies, self-efficacy, and barriers or facilitators to exercising. Participant characteristics and quantitative usability data were descriptively reported. Qualitative data were analyzed using thematic analysis.

**Results:**

A total of 12 PWD tested the usability of the exercise app and completed 96% (69/72) of the usability tasks on the first attempt. Operation speed varied among users, which prompted the development team to make minor revisions to the app. Qualitative results demonstrated 3 overarching themes: *facilitates exercise adoption, positive experiences of videos,* and *easy to learn*. Participants noted that the app circumvented several barriers to exercise associated with leaving the home (eg, inclement weather conditions, exacerbations of health conditions or disability symptoms, difficulties with transportation, and social support).

**Conclusions:**

The mobile exercise app provided a simple platform that was effective, useful, and appreciated by PWD. Participants also perceived the app as easy to use and felt it was a valuable tool for assisting PWD to obtain regular exercise. Study findings also offered insight into the participants’ preferences for mobile exercise apps that can aid future research and development projects. Future exercise trials are needed to determine the true impact of mobile app technology on lifestyle physical activity in people with disabilities.

**Trial Registration:**

Clinicaltrials.gov NCT03024320; https://clinicaltrials.gov/ct2/show/NCT03024320 (Archived by WebCite at http://www.webcitation.org/75hNLgRFH).

## Introduction

According to the United States Census Bureau, 30.6 million people have mobility limitations (eg, difficulty with walking or climbing stairs and wheelchair or cane use) and 15.5 million experience problems with normal activities of daily living [1]. In addition to the direct physical impairments associated with the disability, systematic reviews have identified a multitude of structural and logistical barriers to onsite exercise participation. Some of the more egregious barriers include lack of transportation, extensive time commitment, lack of accessible facilities and equipment, and high cost of a fitness membership [2]. The diversity and number of barriers likely explains why adults with disabilities have higher rates of physical inactivity compared with adults without disabilities. National prevalence data have indicated that 57.4% of adults with mobility limitations living within the United States were inactive (ie, achieving <150 min of moderate-to-vigorous intensity aerobic exercise), compared with only 26.1% of adults without disabilities [3].

Mobile health (mHealth) apps can provide a ubiquitous channel for delivering convenient exercise services to people within their community [4]. In the general adult population, app-based interventions have been found to be efficacious for improving physical activity participation and reducing sedentary behaviors [5]. These apps appeared to be particularly beneficial when accompanied by behavior change techniques. Common examples of these techniques include goal setting, self-monitoring, performance feedback, and social networking [6,7]. Nevertheless, the viability of using mHealth apps for promoting exercise behavior will ultimately depend on participants’ perceptions of their ease of use [8] **.**

Although there are thousands of fitness apps that are commercially available for the general population, few have been developed specifically for people with physical disabilities (PWD) [9]. A survey of 377 people with functional limitations reported that exercise and activity apps were the most commonly used type of mHealth apps [9]. However, only 173 (173/377, 45.8%) of these individuals reported that they could easily locate a suitable app, and the same percent reported that they were satisfied with usability. Within this report, the respondents identified that commercial apps had issues with accessibility and usability and suggested that apps be created with disability-specific content. Although research studies have incorporated self-regulated mHealth apps or Web-based interventions to deliver streamed video content, one-on-one training, and activity tracking and monitoring in PWD, there are limited apps that include customizable exercise content and behavioral change techniques that are tailored to people with a range of functional limitations [10]. Therefore, the purpose of this study was to examine the usability of an inclusive mHealth fitness app that was developed specifically for PWD. The study had 2 aims: (1) quantitatively assess the app’s effectiveness and efficiency and (2) qualitatively explore participants’ satisfaction and usefulness of the app.

## Methods

### Study Design

This study used a nested mixed-methods design (QUAN ->qual) [11] to test the usability of an mHealth fitness app. The design incorporated a primarily quantitative usability study that was followed by a qualitative interview. The study included both quantitative and qualitative data collection to provide an *expanded* evaluation of usability in 4 core areas: *effectiveness, efficiency, satisfaction,* and *usefulness.* These components were selected based upon best practice recommendations for usability testing [8]*.* The study conformed to the Consolidated Standards of Reporting Trials of Electronic and Mobile Health Applications and online Telehealth (CONSORT-EHEALTH) [12].

### Recruitment

Twelve PWD were recruited for this study to satisfy best practice recommendations for usability testing [8].Eligibility criteria included: (1) age 18 to 70 years, (2) documented physical mobility limitation, which we broadly defined as the use of an assistive device as a primary means of mobility or the presence of walking impairments (eg, hemiparesis or drop foot), (3) ability to speak and understand English, and (4) ability to operate an app on a mobile device. Participants were recruited through the network of an internationally recognized community fitness facility which specializes in adapted physical activity programs for PWD. This project was approved by the university institutional review board. Before enrollment, written consent was obtained from each participant.

### Intervention

The mHealth app examined in this study is referred to as Trial #NCT03024320, Scale Up Project Evaluating Response to Home Exercise and Lifestyle Tele-Health (SUPER-HEALTH). This study aimed to test the usability of the SUPER-HEALTH app before evaluating its effectiveness in a randomized controlled trial. The primary component of SUPER-HEALTH was the exercise video content, which included movements that were adapted from a rhythmic movement-to-music (M2M) program for people with multiple sclerosis and stroke. M2M was created and implemented for 4 years at an internationally recognized fitness facility for PWD. SUPER-HEALTH (version 2.3) is a research tool that can be commercially downloaded at no charge, but does require a research team member to activate and tailor the program to meet the functional needs of a participant. The app includes the following features:

- a multicomponent fitness program using a set of videos that can accommodate a variety of functional abilities;
- educational papers with content framed within the social cognitive theory [13];
- the ability to sync and display exercise data from commercial activity monitors;
- achievement rewards (badges);
- social networking functions that include the ability to add other users as friends, communicate with other users via a newsfeed, and private message other users to facilitate social support.

**Figure 1:**
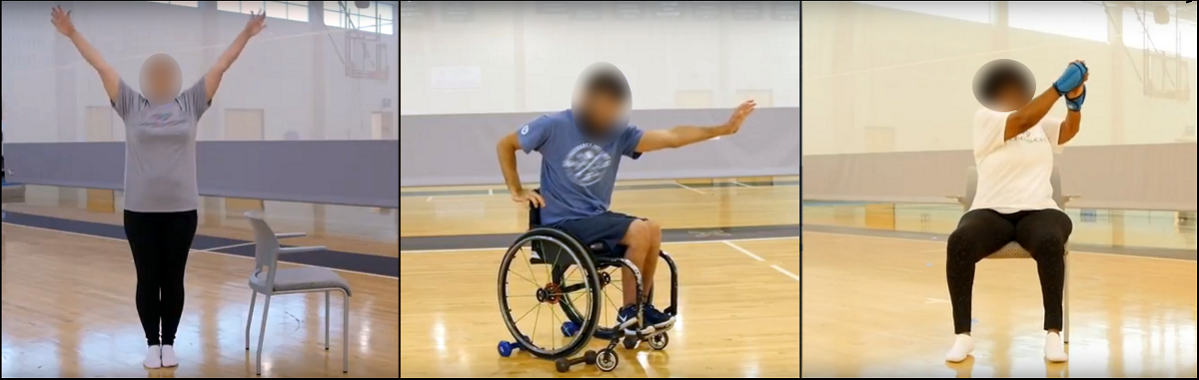
Examples of the 3 different program versions (Left: Version 1; Middle: Version 2; Right: Version 3).

Exercise videos included movements from the M2M program and focused on several components of fitness: range of motion, muscular strength, cardiorespiratory endurance, and functional strength or balance. To be inclusive of a wide variety of functional abilities, the app contained 3 different versions of the M2M program: a version for people with the ability to use the upper and lower limbs, which included movements in the seated and standing position (Level 1); a seated version for people with the ability to use only upper limbs (eg, wheelchair users; Level 2); and a seated and standing version designed for people with the ability to use 1 upper and 1 lower limb (ie, hemiparesis; Level 3). Examples of the movements in the different program versions are shown in Figure 1 **.**

The educational papers in the app included infographics that aimed to enhance self-regulated physical activity behavior and were based on strategies grounded in social cognitive theory [13,14]. These papers targeted 4 core constructs (self-efficacy, goal setting, outcome expectations, and barriers and facilitators), each of which has been recommended to increase physical activity behavior in people with neurologic disabilities [14]. Examples of content included understanding the benefits of physical activity participation in people with disabilities; learning how to monitor physical activity; setting SMART goals (Specific, Measurable, Attainable, Realistic, and Timely); and seeking social support.

### Procedure

This study included 2 phases: usability testing and a qualitative interview. Usability testing included 7 tasks that were followed by a one-on-one semistructured interview, both of which took place at the research laboratory. After obtaining consent, written information including participant demographics (age, sex, race, and education), clinical characteristics (disability or condition and mobility limitation), mobile phone usage, and physical activity status were recorded. Participants were then instructed to login to the app with a standard user account and perform the 7 usability tasks, which included: opening the app; locating and opening a paper; locating and opening a badge; locating, opening, and creating a one-word post on the newsfeed; adding a user as a friend; locating and viewing the leaderboard; and playing the videos and performing the adapted exercise routine. While performing the tasks, the participants used a think aloud approach [8]. A research assistant took written notes while observing participants during the tasks. The assistant also recorded the time it took participants to complete each task, except for the performance of exercise videos.

When instructed to play an exercise video, participants were asked to locate, play, and perform a single video that they felt was suitable to their functional ability. Participants were given access to an archive of videos that included the first 6 weeks of the SUPER-HEALTH 48-week program. The archive included a total of 33 videos that were categorized by the 3 program levels (11 videos per level). Within each program level, there were 4 videos for range of motion exercises, 3 videos for strength, 2 for cardio, and 2 for functional strength. The videos were accompanied by an image that represented the type of movement patterns and positions (seated or standing) that were included within each video. Participants were instructed to perform all movements at a comfortable pace.

After completing the user tasks, participants met with the study investigator and completed a semistructured interview. The interview was conducted in a private setting in the research laboratory. The interview was recorded by an audio device, which was later transcribed for qualitative analysis.

### Measures

#### Summary

App usability was defined in terms of effectiveness (the ease at which individuals can use the product), efficiency (the speed with which an individual can accurately complete a task), usefulness (the extent a product can enable users to achieve their goals and willingness to use the product), and satisfaction (the users’ perceptions and opinions of the product) [8]. Usefulness and satisfaction were explored through qualitative means, whereas effectiveness and efficiency were examined through quantitative metrics.

#### Effectiveness

Research members evaluated the *effectiveness* of users’ experiences with the app by recording the frequency of tasks that participants completed on the first attempt without error or issue. These observations were summed for all users and divided by the total tasks that were completed, which resulted in a single percentage value. The research team set an a priori benchmark of acceptable *effectiveness* at 95% [8].

#### Relative Efficiency

*Relative efficiency* was measured by the time required to complete each of the 6 tasks (video performance task excluded). As the research team anticipated that participants would include older adults, as well as individuals with neurologic and upper-limb impairments, the research team did not set an a priori benchmark to indicate an acceptable level of efficiency. Instead, these data were used to identify problem areas within the app for rectification.

#### Usefulness and Satisfaction

Researchers assessed *usefulness* and *satisfaction* through participants’ qualitative feedback from face-to-face interviews. Each semistructured interview included open-ended questions that sought to gain insight into the participants’ overall perceptions of the app, their likes and dislikes regarding app features and content, whether they would use the app at home, and whether they felt they could find a video that was suitable to their functional ability. The specific interview guide and questions are included in the Multimedia Appendix 1. Two members of the research team conducted the interviews. One interviewer was a research staff member that was trained and supervised by the primary interviewer. The primary interviewer had 4 years of experience with qualitative interviews and had a background in adapted physical activity.

#### Physical Activity

Physical activity status was assessed with the Godin Leisure-Time Exercise Questionnaire (GLTEQ) [15]. The GLTEQ is a questionnaire that asks participants to self-report the number of exercise bouts in a typical week that last longer than 15 min. Bouts are counted for 3 different exercise intensities: light, moderate, and vigorous. Frequency counts for moderate and vigorous intensity exercises are multiplied by 5 and 9, respectively, and summed into a single health contribution score. Activity levels can be compared with the following cut-points: ≥24 sufficiently active, 23-14 moderately active, and <13 insufficiently active [16,17]. This method of scoring has been validated in the general adult population with fair to substantial *k* coefficients for test-retest reliability (*k* coefficient for a 15-day period *=*.65; *k* coefficient for a 30-day period=.45). This scoring method has also been demonstrated to have a moderate correlation (*r*=.46) with moderate-to-vigorous physical activity (measured via accelerometer) in adults with multiple sclerosis [18].

#### Instruments

**Figure 2:**
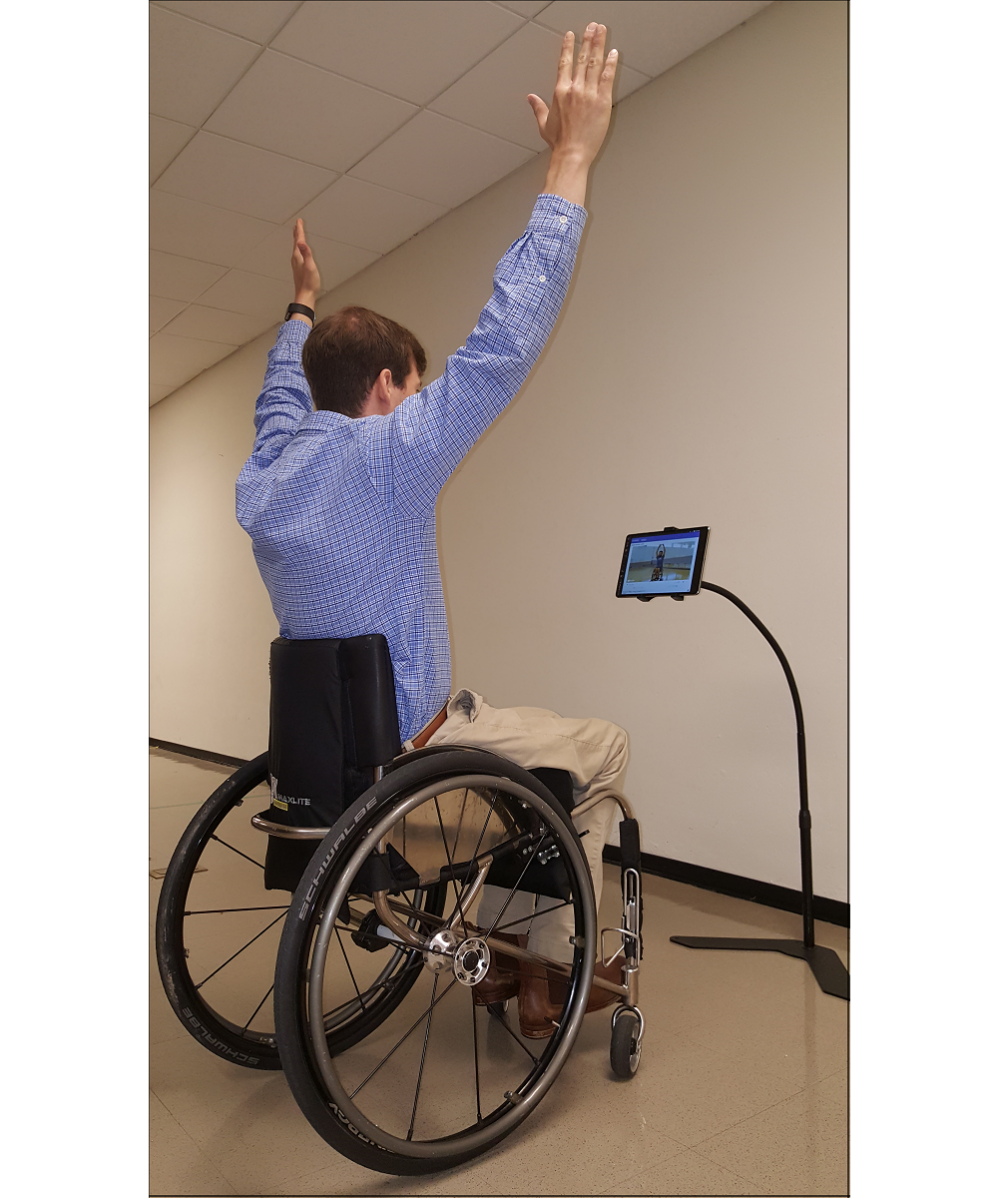
Example of an individual following an exercise routine through a computer tablet mounted to an adjustable stand.

An example of the usability test setup is shown in Figure 2. Equipment included a 10.5-inch Android tablet that came installed with the mHealth fitness app and was mounted to an adjustable floor stand (Standzfree Universal Stand, Standzout).

#### Analysis

The research team’s philosophical assumptions aligned with dialectical pluralism [19]. Within this paradigm, the research team held separate theoretical perspectives for the quantitative and qualitative methods (positivism and interpretivism, respectively). Participant characteristics and quantitative usability data were descriptively reported.

Qualitative data were analyzed using thematic analysis [20], which was underpinned by an interpretivist philosophical approach. Specifically, the analysts ontological beliefs aligned with ontological relativism (ie, reality is multiple and subjective) and their epistemological beliefs with subjectivism (ie, knowledge is socially constructed) [21]**.** In other words, the analysts acknowledged that participants can have multiple explanations for a phenomenon that can be shaped by their backgrounds and interactions with others. Accordingly, the analysts acknowledge that research staff are not blind-observers during the qualitative process: data collection is influenced by the presence and interaction of the interviewer; and themes are interpreted by the analysts and transformed beyond mere explicit statements reported by participants.

The 6 steps proposed by Braun and Clarke [20] were used to guide the thematic analysis process. A total of 2 analysts generated initial codes from segments of a transcribed interview. These codes were then refined into fewer subthemes. The analysts repeated this process for each transcription and evolved their subthemes. The analysts then met to discuss their subthemes, which they then integrated and refined into a single set of themes. These resultant themes were reported. Both analysts had training and experience in mixed-methods research and in developing exercise programs for PWD. One analyst was also the primary interviewer. The other analyst had a physical disability for 12 years.

To enhance the quality of the qualitative research, we adopted a relativist approach that aligned with the ontological and epistemological assumptions that underpinned the qualitative component [22]. First, the qualitative research was aimed at providing a *substantive contribution* [23]*.* This was demonstrated in the results by the efforts to provide meaningful findings that can be used by other investigators, who aim to understand how PWD interact with and respond to exercise technology. Second, *coherence* was sought by using qualitative study procedures throughout the methods and results that fit together and aligned with the goals of the study [24]. Finally, *transparency* was sought by receiving in-depth feedback from a *critical friend* [25], whereby the individual scrutinized matters such as the theoretical preferences, qualitative procedures, and results to encourage reflexivity and alternative explanations and interpretations of the data.

## Results

### Overall

**Figure 3:**
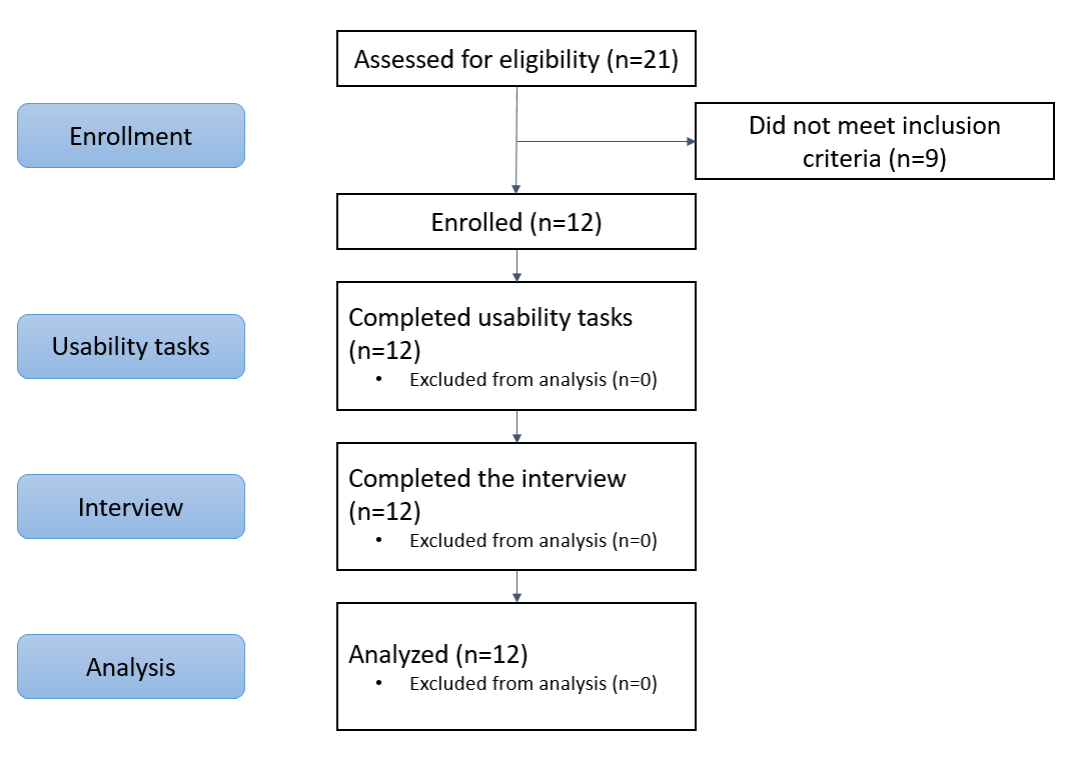
Flow diagram.

Figure 3 displays participants’ progression through each of the study phases. A total of 21 people with disabilities were contacted and screened. Of these, 12 individuals were eligible to participate in the study. Participants had a range of functional limitations.

The study occurred from October 2017 to February 2018. A total of 5 participants performed the standing video set (Level 1), 5 participants performed the seated video set (Level 2), and 2 participants performed the hemiparesis video set (Level 3). Participant demographics and clinical characteristics are shown in Table 1. Each visit took approximately 1.5 hours to complete, which included consent, usability testing and the interview.

### Effectiveness and Efficiency

#### Effectiveness

Participants completed 96% of the usability tasks (69/72) on the first attempt. There were only 3 minor usability issues related to app features. One was because of confusion with wording on the paper’s page and the other 2 were issues with visual cues on the Newsfeed page.

#### Efficiency

Results for the time required to complete each task are shown in Table 2. Developers identified 3 of the 6 tasks that demonstrated substantial variability: locating an earned badge (task 3), locating the Newsfeed page and creating a one-word post (task 4), and adding another user as a friend (task 5).

### Usefulness and Satisfaction

Qualitative results from the semistructured interviews (Textbox 1) demonstrated 3 themes: *facilitates exercise adoption, positive experiences of videos,* and *easy to learn*.

#### Facilitates Exercise Adoption

Participants perceived the app as a powerful and valuable tool for incorporating exercise behavior into the daily activities of individuals with disabilities by circumventing several barriers to exercise associated with leaving the home. Barriers such as inclement weather conditions, exacerbations of health conditions or disability symptoms (eg, arthritis-related inflammation, severe fatigue, and pain), and, most notably, difficulties with transportation (cost, time, and accessibility) were reported by participants. In addition, participants reported that exercising at home could negate feelings of social judgment that occur in a group exercise setting at a fitness facility, such as feelings of conviction from missing an exercise class or embarrassment from poor or incorrect performance in the presence of other class members. To address a need for social support, participants reported that the social media functions within the app provided an opportunity for camaraderie, which could be beneficial for individuals with disabilities who are often isolated within their community. Due to these collective benefits, participants reported that the app was potentially valuable and innovative for inactive people with disabilities who needed to adopt a lifestyle that included regular exercise. As stated by participant 12:

I think that it [the app] is a way of introducing, a way of beginning, and a way of encouraging. My overall impression is that it is something that is needed and something that could lead people out of a more inactive lifestyle...I just think that there are a lot of people like me that need something to start the ball rolling.

Participant 12

**Table 1:** Participant information (N=12).

Characteristic		Value
Age, mean (SD)		52 (15)
**Sex, n**		
	Male	6
	Female	6
**Ethnicity, n**		
	Black	6
	White	6
**Diagnosis, n**		
	Spinal cord injury	5
	Parkinson disease	2
	Arthritis	2
	Multiple sclerosis	1
	Stroke	1
	Traumatic brain injury	1
**Mobility device or limitation, n**		
	Manual wheelchair	4
	Walker	2
	Cane	1
	Cane and hemiparesis	1
	Hemiparesis	1
	Orthotic device	1
	Poor balance	1
	Power wheelchair	1
GLTEQ^a^: Health contribution score, mean (SD)		34.7 (28)
GLTEQ: Sufficiently active (score ≥23), n		7
GLTEQ: Moderately active (score 14-23), n		2
GLTEQ: Insufficiently active (score <13), n		3
**Mobile phone users, n**		
	Yes	10
	No	2
**Education, n**		
	Graduate degree	5
	College graduate	5
	Some college	2

^a^GLTEQ: Godin Leisure Time Exercise Questionnaire.

**Table 2:** Efficiency results.

Usability tasks	Median time to completion (Interquartile range), seconds	CI, seconds
Task 1: Open the menu	3 (9.2)	2.5-10.4
Task 2: Locate and open a paper	8.9 (6.6)	7.2-15.0
Task 3: Locate a badge	21 (7.5)	16.5-23.9
Task 4: Newsfeed one-word post	37.5 (26.3)	23.4-42.6
Task 5: Add a user as a friend	12 (24.8)	9.1-31.1
Task 6: Locate and view the leaderboard	6 (2.1)	4.3-6.63

**Qualitative themes and subthemes.**

Facilitates exercise adoption

- Circumvents exercise barriers
- Reach and Impact
- Opportunities for social support
- Innovative

Positive video experience

- Mode of delivery
- Exercise movements

Easy to learn

- Intuitive
- Short learning curve

#### Positive Experiences of Videos

Participants held favorable views of how the exercise videos were packaged and delivered. In addition to having control over the exercise environment (ie, the home setting), participants also experienced a sense of control over the pace of the exercise sessions, which was primarily because of the ability to start and stop videos at their convenience. One participant commented:

I felt like I was in charge, as far as, getting it, doing it, and stopping when I need to.

Participant 9

Moreover, while performing the videos, participants felt as if they were engaged in a real-time group exercise class because of the enthusiastic and engaging mannerisms and rhetoric provided by the exercise instructor:

She [the instructor] said you could go at your own pace...Even though it was not back and forth communication, it was just the way she talked to you; she wasn’t condescending.

Participant 4

Participants also acknowledged a high level of appreciation for the exercise movements. All participants found videos that contained movements that were suitable to their functional ability and reported that the movements were potentially inclusive for a large variety of individuals with disabilities. Participants also noted favorable perceptions of the novelty, variety, and perceived health benefits of the movements, along with the comfortable pace and nonjudgmental manner to which they were guided.

#### Easy to Learn

Participants identified that the app was *easy to learn.* When asked how confident they felt using the app independently, participants reported high levels of confidence. This was because of the ease at which the app could be navigated and operated, as well as the similarity of the app user interface compared with other available apps in the marketplace. However, participants did mention that efficient use of the app would require a learning curve, which could be achieved independently with brief instructions before use.

## Discussion

### Summary

Multiple barriers must be overcome for many PWD to engage in regular exercise. For this reason, we designed an app that provides high quality exercise videos that are customized for people with a range of physical disabilities and can be accessed in the home setting through a tablet. The usability of this system was tested through separate quantitative and qualitative assessments. These findings are discussed in an integrated format to provide a holistic evaluation of usability. Overall, the findings demonstrated that the SUPER-HEALTH app was effective, valuable, and useful for people with disabilities.

### Quantitative Findings

Quantitative results demonstrated that a variety of PWD could successfully operate the SUPER-HEALTH app. The high percentage of successful users (>95%) on their first attempt achieved our a priori criteria for acceptable effectiveness. The only minor issue related to efficiency was the time taken for completion of the 3 tasks (adding another user as a friend, locating an earned badge, and creating a post on the Newsfeed). This informed the development team that improvements could still be made to certain visual aspects of the app.

### Qualitative Findings

The qualitative results demonstrated overtly positive themes related to usefulness and satisfaction. Combined with inclusive exercise program versions, opportunities for social support, and an intuitive user interface, participants reported that the app was potentially valuable for themselves and the general population of people with disabilities who desire to start an exercise program. Specifically, the app was considered an innovative and convenient alternative to onsite exercise at a fitness facility because it circumvented several barriers (eg, weather, transportation, health conditions or symptoms). These barriers are consistent with those reported in the extant literature for PWD [2].

### Integrated Findings

On the basis of the integrated quantitative and qualitative findings, the research team addressed 2 critical development questions: (1) Were further revisions to the app necessary? and (2) Were further usability tests necessary? Due to the positive qualitative feedback and effectiveness findings, the research team collectively agreed that no further usability tests were necessary. However, based on efficiency data and the *easy to learn* theme, the researchers informed the development team to add minor visual improvements to app version 2.5.1 (eg, increased size of buttons and fonts and alterations to wording and color) and provide future participants or users with more detailed instructions before tablet use.

### Future Studies

In addition to testing the usability of the SUPER-HEALTH app, study findings provide a foundation for researchers and developers who aim to tailor exercise apps or similar Web-based programs for adults with physical disabilities. Qualitative findings suggested that PWD perceive several benefits to self-regulated Web-based exercise programs. First, individuals appreciate a sense of control over the pace of exercise sessions, which provides ample time to learn new movements and take breaks when necessary. Second, some individuals who are hesitant about exercise in a public setting because of perceived judgment from others, might prefer app-based exercise in the home setting versus onsite exercise at a facility. Last, participants reported that an exercise app should not replace physical activity participation within the community such as exercising at a nearby facility or park. Instead, participants noted that the end goal for exercise-based apps should be to transition people from exercising in the home setting to physical activity within their community.

### Limitations

This study had a few limitations. First, although a sample size of 12 may be sufficient for detecting usability issues, the generalizability of these findings is limited to individuals with similar conditions, impairments, or characteristics (eg, education level, ethnicity, and technology proficiency) as our study participants. Second, most participants were active exercisers recruited through the network of an internationally recognized fitness facility, and therefore may not represent the larger population of individuals with disabilities, including those who are inactive. However, we felt the inclusion of active individuals provided rich insight on both exercise adoption (ie, beginning participation in exercise) and long-term participation (ie, sustainability). We also did not want to exclude individuals who could potentially benefit from participation in the larger trial. Third, a few individuals showed symptoms of cognitive impairment that could have explained the variability observed in efficiency data, but cognitive impairment was not assessed by the research staff. Fourth, the study did not include an even distribution of participants among the 3 exercise video levels and only examined the usability of select videos within the program, which warrants an examination of program feasibility.

### Conclusions

This study demonstrated that the SUPER-HEALTH app provides a simple platform that can be easily operated by a wide variety of users with physical disabilities. Study findings also provide insight into participants’ preferences for mobile exercise apps that can inform future research and development projects. Future research should examine app feasibility in the real-world setting (ie, home) to provide further insight into the app’s usability before implementation in an exercise intervention.

## Appendix

Multimedia Appendix 1 Qualitative interview script.

## Abbreviations

GLTEQ: Godin Leisure-Time Exercise Questionnaire
mHealth: mobile health
M2M: movement-to-music
PWD: people with physical disabilities
SUPER-HEALTH: Scale Up Project Evaluating Responsiveness to Home Exercise and Lifestyle Tele-Health
