# Supplementary material for: A Mobile App to Promote Adapted Exercise and Social Networking for People With Physical Disabilities: Usability Study
Source: JMIR Form Res. 2019 Mar 19;3(1):e11689. doi: 10.2196/11689 (PMC6444218; doi:10.2196/11689)
Supplement: Multimedia Appendix 1 [file formative_v3i1e11689_app1.pdf]

Appendix:

<u>Qualitative Interview:</u> Audio Recorded for Transcription (Semi-structured; Open-ended questions; Comfortable Setting for the Participant; Researcher's Philosophical Assumptions Framed within Interpretivism)

Interview Briefing Script (start audio recording before reading script):

1. "You have been asked to participate in an interview to provide your overall experience of the app. We are researchers from UAB and Lakeshore Foundation. This interview should take no longer than 30 minutes. Should you agree to participate, the results of these findings will be used in a future research study. This interview will be recorded via audio device and can be performed in a comfortable setting of your choosing. Do you agree or disagree to participate in the interview?"

2. *If they agree*, then say: "Okay, before we get started, please try to be open and honest about your responses and try not to let my involvement influence how you answer a question. After we finish this interview, we may contact you in the future if we have additional questions. Do you have any questions before we begin?"

Interview Questions: (Prompts: "Could you tell me more about that?", "How did that make you feel?", "Do you have anything else you'd like to add about that?")

1. As an icebreaker, what do you like to do on your free time?
2. Could you please describe to me your overall perceptions of using the app?
3. What did you like about the app?
4. What did you dislike about the app?
5. How confident are you that you could operate the app by yourself?
6. Would this app you be something you would be open to using?
7. Were you able to find an exercise video that was suitable to your functional ability?
